# MRI findings of knee abnormalities in adolescent and adult volleyball players

**DOI:** 10.1186/s40634-017-0080-x

**Published:** 2017-02-21

**Authors:** Heide Boeth, Aoife MacMahon, Felix Eckstein, Gerd Diederichs, Arne Schlausch, Wolfgang Wirth, Georg N. Duda

**Affiliations:** Julius Wolff Institute, Charité - Universitätsmedizin Berlin, Berlin, Germany

**Keywords:** Magnetic resonance imaging, Osteoarthritis, Athletes, Volleyball, Knee abnormalities, Adults, Adolescents, Males, Females, Age, Sex

## Abstract

**Background:**

To longitudinally and cross-sectionally evaluate knee abnormalities by sex and age in adolescent and adult volleyball athletes over 2 years using magnetic resonance imaging (MRI).

**Methods:**

Thirty-six high-level volleyball athletes (18 adolescents: 56% female, mean age 16.0 ± 0.8 years; and 18 adults: 50% female, mean age 46.8 ± 5.1 years) were imaged by MRI at BL and at 2-year follow-up (FU). Prevalence and severity of cartilage lesions, subarticular bone marrow lesions (BMLs), subarticular cysts, osteophytes, and ligament and meniscus integrity were evaluated by sex and by age cohort (adolescents and adults) using the whole-organ MRI score (WORMS).

**Results:**

There were no significant longitudinal changes in any of the features within any of the sex or age groups. No significant differences were found in overall prevalence or severity of any of the features between males and females, although at FU, males had a significantly higher prevalence of osteophytes in the medial femorotibial joint (MFTJ) than females (*p*=0.044). Compared to adolescents, adult volleyball players had a significantly greater prevalence and severity of cartilage lesions (*p*<0.001 for both), BMLs (*p*=0.0153 and *p*=0.005), and osteophytes (*p*≤0.003 and *p*<0.001), and more severe meniscal lesions (*p*≤0.021).

**Conclusion:**

We found significant differences in the prevalence and severity of knee abnormalities between adolescent and adult volleyball players, but no overall differences by sex. These findings lay the groundwork for further investigations with larger cohorts and longer FU times to determine whether or not these knee abnormalities are associated with the development of OA.

## Background

Primary osteoarthritis (OA) is the most common form of arthritis, accounts for more mobility disability than any other disease (Felson 2014), and is the second greatest cause of disability in the world (Conaghan et al. 2014). Knee OA is more prevalent than other types of OA and is also more commonly seen in younger age groups (Oliveria et al. 1995, Bliddal and Christensen 2009). Studies have shown that high impact sports such as volleyball, characterized by short intensive and explosive actions (Mroczek et al. 2014), can lead to OA (Marti et al. 1989, Kujala et al. 1994).

Magnetic resonance imaging (MRI) is uniquely suited to image articular tissues, including cartilage, menisci, and ligaments, and can thus provide greater insight into the development of pathology in the whole joint (Peterfy et al. 2004). Several prior studies have utilized MRI to evaluate the knees of asymptomatic athletes. These studies have found wide ranges in the prevalence of knee abnormalities, including in adult basketball players (Major and Helms 2002, Kaplan et al. 2005, Walczak et al. 2008), gymnasts (Ludman et al. 1999), and marathon runners (Stahl et al. 2008). One MRI study of asymptomatic adolescent soccer players found that they had more knee abnormalities than matched controls (Soder et al. 2011). However, knee abnormalities in adolescent and adult volleyball athletes have not previously been investigated with MRI.

Before the age of 50 years, OA has a greater prevalence in males, but after the age of 50 years, females show a higher prevalence of hand, foot and knee OA (Felson et al. 2000). Yet, the role of sex in knee OA among athletes of high-impact sports remains unclear. It has been suggested that female soccer players have a higher incidence of post-injury OA than male soccer players due to the former’s higher rate of anterior cruciate ligament (ACL) injuries in that sport (Lohmander et al. 2004). In contrast, a meta-analysis concluded that females and males were equally likely to develop OA following ACL reconstruction (Tan et al. 2015). Thus, uncertainty remains regarding the influence of sex on OA and knee pathology in athletes.

It has been suggested that baseline (BL) MRI knee studies can be clinically utilized to prevent injury and improve diagnostic accuracy in athletes, which could reduce loss of playing time. In addition, when symptoms do arise, BL MRI screenings can be compared with those obtained at follow-up to identify new knee abnormalities or lesions more likely to be correlated with symptoms (Walczak et al. 2008). Moreover, MRI screenings may aid in the understanding of knee abnormalities found in athletes who subject the knee to high loads and are at high risk of subsequent OA. Thus, the aim of this study was to longitudinally and cross-sectionally evaluate knee abnormalities by sex and age in adolescent and adult volleyball athletes over 2 years using MRI. These findings can help elucidate the role of MRI in aiding clinical diagnosis and in the prevention and treatment of injuries in athletes.

## Methods

### Subjects

The study protocol was approved by the local ethics committee and all participants (and/or their parents) had signed informed consent to participate in the study. We studied a convenience sample of 36 high-level volleyball athletes, 18 adolescents (8 male, 10 female, mean age 16.0 ± 0.8 years, body mass index (BMI): 21.50 ± 1.79 kg/m^2^) and 18 adults (9 male, 9 female, BL age 46.8 ± 5.1 years, BMI: 24.41 ± 2.91 kg/m^2^) at BL and at 2-year follow-up (FU) (Eckstein et al. 2014). As the aim was to include high-level volleyball athletes who competed or had competed at the national level, only a small cohort was available for study. The inclusion criteria for adolescents were age less than or equal to 17 years, current participation in the 2-year volleyball training program at Olympiastützpunkt Berlin, and prior volleyball participation in a club for at least 3 years. The volleyball training program at Olympiastützpunkt Berlin consisted of 2-h training sessions twice daily, 6 days a week (24 h per week), for 2 years. Inclusion criteria for the adults were age greater than 40 years, past participation in the volleyball training program at Olympiastützpunkt Berlin, and continued volleyball participation since then of at least 2 h a day, twice a week (4 h per week). Exclusion criteria for both groups were participation in other sports or knee injury requiring surgery less than 1 year prior to the BL evaluation.

Prior knee injuries which required surgical intervention were documented at BL through self-reported questionnaires. Eight out of the 18 adult volleyball players (44%) had a prior knee injury. Among the adult males, 1 had a lateral meniscectomy, 1 had medial and lateral meniscectomies, 1 had an unspecified meniscectomy, and 1 had other prior knee surgery. Among the adult females, 1 had a patella surgery, 1 had a knee arthroscopy, 1 had a meniscus surgery, and 1 had a medial meniscectomy. Among the adolescent volleyball players, there was one prior knee injury in a female (5%), which was an ACL rupture that had been treated with ACL reconstruction. All prior knee injuries occurred in the athlete’s take-off leg.

### Study design

The design of this study addresses four main questions, which are 1) changes in prevalence and severity of knee abnormalities over time, 2) differences in knee abnormalities by sex, 3) differences in severity of knee abnormalities by age group, and 4) the impact of prior knee injuries on prevalence and severity of knee abnormalities.

### MRI and grading of articular tissue pathology

**Fig. 1:**
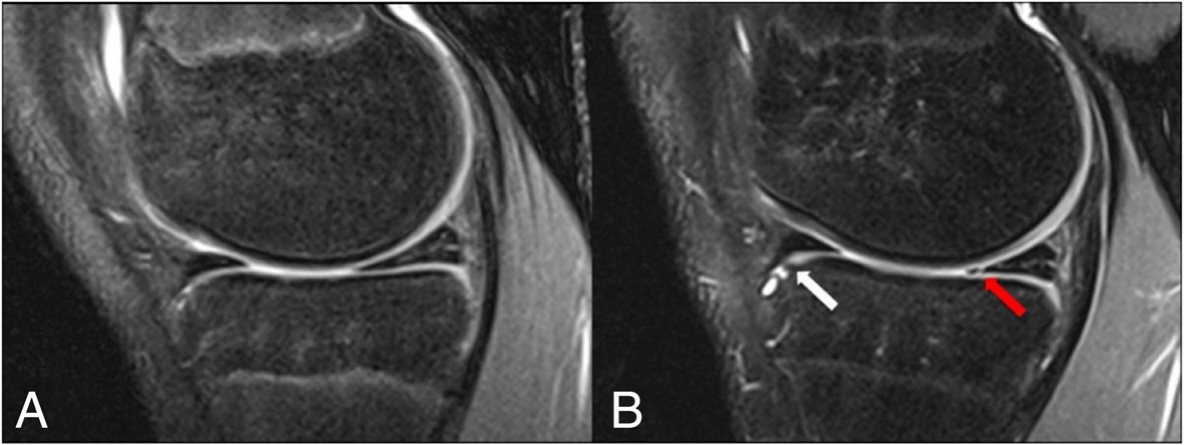
Sagittal PD FS images showing the medial compartment of the femorotibial joint of an adolescent male volleyball player at baseline **a** and 2- year follow-up **b**. In the follow-up scan, there is a new osteophyte at the anterior cortex of the tibial plateau (*white arrow*) and a new *vertical* tear of the posterior horn of the meniscus (*red arrow*), which were not visible in the baseline scan

MRI was performed in a 1.5 Tesla *Avanto* scanner (Siemens Medical Systems, Erlangen, Germany) using a dedicated 8-channel knee coil. The take-off leg was imaged in all participants in supine position with the leg in full extension. A 2D coronal proton-density (PD) weighted turbo spin-echo MR sequence with fat suppression (0.4167 × 0.4167 mm in-plane resolution, 3 mm slice thickness, 3.6 mm slice spacing, 29 ms echo time, 3520 ms repetition time, 150° flip angle) as well as a 3D axial T2-weighted Multi-Echo Data Image Combination (MEDIC) sequence (0.167 × 0.167 mm in-plane resolution, 1.2 mm slice thickness, 21 ms echo time, 38 ms repetition time, 8° flip angle) were used for imaging at both BL and FU scans (blinded citation). Fig. 1 shows an example of the sagittal PD fat suppression images of the femorotibial joint of a male volleyball player at BL (A) and FU (B).

All images were assessed on a picture archiving communications system (PACS) by a radiologist with 12 years of experience in musculoskeletal imaging. The radiologist was blinded to the clinical information of each subject and to the time point of the MRI.

### Quantification of knee joint disability

To analyze structural pathology of the knees, the whole-organ magnetic resonance imaging score (WORMS) was used (Peterfy et al. 2004). Cartilage status, subarticular bone marrow lesions (BMLs), subarticular cysts, osteophytes, and ligament and meniscus integrity were assessed using the WORMS scoring system. The first four features were chosen because they related to the articular surfaces, and the last two were included due to their role in cartilage loss and OA (Peterfy et al. 2004). The investigated regions of the knee included the medial femorotibial joint (MFTJ), lateral femorotibial joint (LFTJ), and patellofemoral joint (PFJ). Region subspinous (S), which is a non-articulating portion of the tibial plateau, was excluded, such that 14 rather than the originally described 15 regions were assessed. Detailed information about the subdivisions into 15 different regions are given in Peterfy et al. (Peterfy et al. 2004). Each assessed feature and its respective WORMS scoring system is shown in Table 1.

**Table 1:** Assessed features of the WORMS scoring system and their respective subscales

Feature	Score
Cartilage status	0 = normal thickness and signal 1 = normal thickness but increased signal on T2-weighted images 2.0 = partial-thickness focal defect <1 cm at greatest width 2.5 = full-thickness focal defect <1 cm at greatest width 3 = multiple areas of partial-thickness (grade 2.0) defects intermixed with areas of normal thickness, or a grade 2.0 defect wider than 1 cm but <75% of the region 4 = diffuse (≥75% of the region) partial-thickness loss 5 = multiple areas of full-thickness loss (grade 2.5) or a grade 2.5 lesion wider than 1 cm but <75% of the region 6 = diffuse (<75% of the region) full-thickness loss
Subarticular BMLs	0 = none 1 = <25% of the region 2 = 25 to 50% of the region 3= > 50% of the region
Subarticular cysts	0 = none 1 = <25% of the region 2 = 25 to 50% of the region 3= > 50% of the region
Osteophytes	0 = none 1 = equivocal 2 = small 3 = small-moderate 4 = moderate 5 = moderate-large 6 = large 7 = very large
Anterior and posterior cruciate ligament and medial and lateral collateral ligaments	0 = intact 1 = torn A total ligament score was calculated by adding the sum of cruciate ligaments and half of the sum of collateral ligaments. Cruciate ligament damage was given greater weight to account for its higher significance in functional impairment of the knee.
Lesions of the medial and lateral menisci	0 = intact 1 = minor radial tear or parrot-beak tear 2 = non-displaced tear or prior surgical tear 3 = displaced tear or partial resection 4 = complete maceration/destruction or complete resection
Overall meniscus integrity	0 = all 0 1 = at least 1, but no >1 2 = 2 in only one region 3 = 2 in more than one region 4 = 3 in one or more regions 5 = 4 in only one region 6 = 4 in more than one region.

The prevalence of each WORMS feature was assessed as follows. For each subject, a feature was considered present for a WORMS grade ≥1 and absent for WORMS grade = 0 with the exception of cartilage lesions and osteophytes, which were considered present for a WORMS grade ≥ 2 and absent for a WORMS grade ≤1.

When comparing the severity of MRI abnormalities between groups, cartilage abnormalities included cartilage WORMS scores = 1 (Table 1).

### Statistics

Subjects were stratified by sex and by age (adolescents and adults) cohort. Since only one adolescent had a prior knee injury that required surgical repair, the effect of prior knee injuries that required surgical intervention on prevalence and severity of knee pathology was assessed only within the adult cohort. Prevalences of each feature are reported as percentages, and severities are reported as medians and interquartile ranges (IQRs) of all abnormalities, i.e. of WORMS scores of subjects with a score ≥ 1 for a given feature, unless stated otherwise. Fisher’s exact tests were used to compare differences in prevalence and Mann Whitney U tests were used to compare differences in severity of pathological knee features by age, sex, prior knee injury, and knee region. McNemar’s tests were used to compare differences in prevalence and Wilcoxon signed-rank tests were used to compare differences in severity of knee pathology from BL to FU within age and sex groups. For all tests, a *p*-value < 0.05 was considered significant. No adjustment for multiple analyses was made, as this was an exploratory study which aimed to identify preliminary results that could undergo more statistically rigorous future investigations. All statistical analyses were conducted using SPSS Statistics 18 (IBM SPSS Statistics, USA).

## Results

### Longitudinal changes in prevalence and severity of knee abnormalities

There were no significant changes found in the prevalence or severity of any of the examined knee features from BL to FU in any of the groups by sex (*p* ≥ 0.216 and *p* ≥ 0.078 respectively) or age (*p* ≥ 0.25 and *p* ≥ 0.058 respectively). At FU, one additional male adult had a cartilage lesion, three additional adolescent males had osteophytes, and one additional adult male had a meniscal lesion. Two additional adult females had BMLs at FU, and one additional adult female had osteophytes. All values cited forthwith refer to BL values unless stated otherwise.

### Differences in knee abnormalities by sex

No significant differences were found between sexes in prevalence of any of the knee features assessed (Table 2). At FU, males had a significantly higher proportion of osteophytes in the MFTJ (12/17, 71%) than females (6/19, 32%; *p* = 0.019), but this did not reach statistical significance at BL (males: 11/17, 65%; females: 6/19, 32%; *p* = 0.093). The only ligamentous lesions observed were one torn ACL and one torn lateral collateral ligament, both in adult males.

**Table 2:** Prevalence of knee abnormalities in subjects stratified by sex. Cartilage lesions and osteophytes were considered present for a WORMS score ≥ 2, and all other features were considered present for a WORMS score ≥ 1. Data presented as number of subjects (percentage of group)

	Baseline			Follow-up		
	Males (*n* = 17)	Females (*n* = 19)	*p*-value	Males (*n* = 17)	Females (*n* = 19)	*p*-value
Cartilage lesions	6 (35)	4 (21)	0.463	6 (35)	4 (21)	0.463
Bone marrow lesions	7 (41)	5 (26)	0.483	7 (41)	7 (37)	-
Subarticular cysts	4 (24)	3 (16)	0.684	4 (24)	3 (16)	0.684
Osteophytes	12 (71)	12 (63)	0.732	15 (88)	13 (68)	0.236
Ligamentous lesions	2 (12)	0 (0)	0.216	2 (12)	0 (0)	0.216
Meniscal lesions	6 (35)	9 (47)	0.516	7 (41)	9 (47)	0.749

There were no statistically significant differences in severity of overall knee abnormalities between sexes (Table 3). However, cartilage abnormalities, BMLs, and osteophytes tended to be worse in males than in females. There were no significant differences in severity of knee abnormalities between sexes found within any of the specific regions assessed.

**Table 3:** Severity of knee abnormalities in subjects stratified by sex. Severity presented as median (interquartile range) for subjects with any abnormality, i.e. WORMS score ≥ 1, for each feature. *P*-values are from Mann Whitney U tests of scores from all subjects in each sex group

	Baseline					Follow-up				
	Males (*n* = 17)		Females (*n* = 19)			Males (*n* = 17)		Females (*n* = 19)		
	*n*	Severity	*n*	Severity	*p*-value	*n*	Severity	*n*	Severity	*p*-value
Cartilage abnormalities	6	5 (4.3, 5)	6	2.8 (1.4, 3)	0.438	7	5 (4.5, 5.5)	6	3 (1.5, 4.5)	0.328
Bone marrow lesions	7	2 (1.5, 2)	5	1 (1, 1)	0.204	7	2 (1.5, 3)	7	1 (1, 1.5)	0.457
Subarticular cysts	4	1.5 (1, 2)	3	1 (1,2)	0.535	4	1.5 (1, 2)	3	1 (1, 1.5)	0.535
Osteophytes	15	3 (2, 4)	19	2 (1, 2)	0.462	15	2 (2, 4.5)	19	2 (1, 2.5)	0.279
Ligamentous lesions	2	0.8 (0.6, 0.9)	0	–	0.129	2	0.8 (0.6, 0.9)	0	–	0.129
Meniscal lesions	6	1.5 (1, 2.8)	9	2 (1, 2)	0.51	7	2 (1, 2)	9	2 (1, 2)	0.843

### Differences in severity of knee abnormalities by age

Adults had a significantly greater prevalence of cartilage lesions and osteophytes than adolescents (*p* < 0.001 and *p* = 0.001 respectively; Table 4). Cartilage lesions were particularly more prevalent in the MFTJ and PFJ in adults (7/18, 39% for each) compared to adolescents (0/18, 0% for each; *p* = 0.008). Osteophytes were more prevalent in all regions assessed in adults compared to adolescents (MFTJ: *p* = 0.001; LFTJ and PFJ: *p* = 0.002). Lateral meniscal lesions were especially more prevalent in adults (BL: 6/18, 33%, FU: 7/39, 39%) than in adolescents (BL: 0/18, 0%, FU: 1/18, 6%; *p* = 0.019 and *p* = 0.041, respectively).

**Table 4:** Prevalence of knee abnormalities in subjects stratified by age group. Cartilage lesions and osteophytes were considered present for a WORMS score ≥ 2, and all other features were considered present for a WORMS score ≥ 1. Data presented as number of subjects (percentage of group)

	Baseline			Follow-up		
	Adolescents (*n* = 18)	Adults (*n* = 18)	*p*-value	Adolescents (*n* = 18)	Adults (*n* = 18)	*p*-value
Cartilage lesions	0 (0)	10 (56)	**<0.001**	0 (0)	10 (56)	**<0.001**
Bone marrow lesions	4 (22)	8 (44)	0.289	3 (17)	11 (61)	**0.0153**
Subarticular cysts	2 (11)	5 (28)	0.402	2 (11)	5 (28)	0.402
Osteophytes	7 (39)	17 (94)	**0.001**	10 (56)	18 (100)	**0.003**
Ligamentous lesions	0 (0)	2 (11)	0.486	0 (0)	2 (11)	0.486
Meniscal lesions	5 (28)	10 (56)	0.176	5 (28)	11 (61)	0.0922

*P*-values presented in bold capture demonstrate significance (*p* < 0.05)

Adults had significantly worse osteophytes (*p* < 0.001) and meniscal status (*p* = 0.021) than adolescents. BMLs were also more severe in adults than in adolescents, but this was only statistically significant at FU (BL: *p* = 0.107, FU: *p* = 0.005) (Table 5). The higher severity of cartilage lesions in adults than adolescents was seen in all regions assessed (MFTJ: *p* = 0.004, LFTJ: *p* = 0.018, PFJ: *p* = 0.001), which was also the case for osteophytes (*p* < 0.001 for MFTJ, LFTJ, and PFJ). Lateral meniscal lesions were also significantly worse in adults than in adolescents (*p =* 0.008).

**Table 5:** Severity of knee abnormalities in subjects stratified by age group. Severity presented as median (interquartile range) for subjects with any abnormality, i.e. WORMS score ≥ 1, for each feature. *P*-values are from Mann-Whitney U tests of scores from all subjects in each age group

	Baseline					Follow-up				
	Adolescents (*n* = 18)		Adults (*n* = 18)			Adolescents (*n* = 18)		Adults (*n* = 18)		
	*n*	Severity	*n*	Severity	*p*-value	*n*	Severity	*n*	Severity	*p*-value
Cartilage abnormalities	0	–	12	3 (2.5, 5)	–	0	–	13	3 (3, 5)	–
Bone marrow lesions	4	1 (1, 3)	8	2 (1, 2)	0.107	3	1 (1, 1.5)	11	2 (1, 2.5)	**0.005**
Subarticular cysts	2	1 (1, 1)	5	2 (1, 2)	0.169	2	1 (1, 1)	5	2 (1, 2)	0.169
Osteophytes	16	1 (1, 2)	18	3 (2, 5)	**<0.001**	16	2 (1, 2)	18	2.5 (2, 5)	**<0.001**
Ligamentous lesions	0	–	2	1 (1, 1)	0.151	0	–	2	1 (1, 1)	0.151
Meniscal lesions	5	1 (1, 1)	10	2 (2, 3)	**0.021**	5	1 (1, 1.3)	11	2 (2, 3.5)	**0.015**

*P*-values presented in bold capture demonstrate significance (*p* < 0.05)

### Impact of prior knee injuries on prevalence and severity of knee abnormalities

Adults with prior knee injury had a greater prevalence of meniscal lesions than those without prior knee injury at BL (7/8, 88% vs. 3/10, 30%; *p* = 0.025) and FU (7/8, 88% vs. 4/10, 40%; *p* = 0.066). Adults with prior knee injury had more severe meniscal lesions than those without prior knee injury at BL (2 (1.8, 2.3) vs. 0 (0, 0.8) for all subjects in each group; *p* = 0.015) and FU (2 (1.8, 4) vs. 0 (0, 1.8) for all subjects in each group; *p* = 0.029). At FU, adults with prior knee injury had more severe osteophytes than adults without prior knee injury (5 (4.3, 5.3) vs. 3 (2, 3.8) for all subjects in each group, respectively; *p* = 0.043), but this did not reach significance at BL (5 (3.5, 5) vs. 3 (2, 3) for all subjects in each group, respectively; *p* = 0.091). No other significant differences were found in prevalence or severity of knee abnormalities between adults with and without prior knee injuries.

## Discussion

In this cross-sectional and longitudinal study, we aimed to investigate knee abnormalities in adolescent and adult high-level volleyball athletes using MRI. Our main findings were that over half of the adults had cartilage and meniscal lesions, and all demonstrated osteophytes. Adults had a higher prevalence and severity of cartilage lesions, osteophytes, BMLs, and more severe meniscal lesions than adolescents. We found no significant differences between males and females in the overall prevalence or severity of knee abnormalities, although at FU, males showed a higher rate of osteophytes in the MFTJ than females. There were no BL to FU changes in prevalence or severity of knee abnormalities in any of the groups,

It is surprising that we found no significant longitudinal changes in prevalence or severity of knee abnormalities within any of the age or sex groups. Ding et al. found both increases and decreases in the severity of knee cartilage defects and osteophytes within 2 years in a cohort with a mean age of 45 years and a 17% incidence of knee OA (Ding et al. 2006). Wang et al. also found that knee cartilage defects tended to progress over 2 years in normal subjects with a mean age of 55.6 years (Wang et al. 2006). The relatively young age of our cohort may explain the lack of longitudinal changes we observed; however, future studies with larger cohorts and longer FU time are required to confirm this.

Cartilage lesions were present in 56% of the adult volleyball athletes at BL and FU, but in none of the adolescents. The lack of cartilage lesions found in the adolescents is in accordance with a previous MRI study of adolescent soccer players, in which no cartilage lesions were found (Soder et al. 2011). Major et al. found that 41% of 34 knees of varsity college basketball players showed cartilage abnormalities (Major and Helms 2002), and Kaplan et al. found that 47.5% of 40 knees of professional basketball players had cartilage lesions (Kaplan et al. 2005), both lower rates than those found in our study. One reason for this may be that 44% of the adults in our study had undergone prior knee surgery, which increased their risk of cartilage lesions (Nepple et al. 2012). However, they were still able to participate in a high level of volleyball.

Osteophytes are fibrocartilaginous and skeletal outgrowths that are central to OA pathophysiology (Peterfy 2003, Goldring and Goldring 2010). They are found in the margins of the knee, at areas of joint loading, which strongly suggests that mechanical factors contribute to their formation (Goldring and Goldring 2010). Our finding that males had a higher rate of osteophytes in the MFTJ than females at FU may be explained by previous findings that females show greater knee valgus than males during landing (Kernozek et al. 2005, Holden et al. 2015). Since valgus alignment decreases the stress on the medial side of the tibiofemoral joint (Sharma et al. 2001), this may cause female volleyball athletes to experience decreased stress on the MFTJ during landing, resulting in the development of fewer osteophytes in that region. This may reflect regional differences in long-term risk of knee OA, as valgus alignment has been associated with a nearly 5-fold increase in lateral progression, and varus alignment with a 4-fold increase in medial progression of knee OA (Sharma et al. 2001). Our findings lay the groundwork for future studies to investigate this regional difference in osteophyte formation between males and females with larger cohorts and longer-term FU. Such studies may also clarify whether the trends we observed of more severe cartilage abnormalities, BMLs, and osteophytes in males are meaningful.

Previous studies have generated mixed results regarding differences in OA-related knee abnormalities between males and females as determined by MRI. In a study by Ding et al. of 372 subjects with a mean age of 45 years, males had higher cartilage volume than females at all sites (Ding et al. 2003). In another study in the same cohort, females showed a greater decrease in knee cartilage volume than males over 2 years, which first became apparent at age 40 and became more pronounced with increasing age (Ding et al. 2007). Wang et al. found in healthy subjects that knee cartilage defects were more likely to progress in males than females over 2 years (Wang et al. 2006). None of these studies investigated BMLs, subarticular cysts, osteophytes, or ligamentous or meniscal lesions. However, a study in long-distance runners with a mean age of 33 years found no difference between males and females in the prevalence of chronic knee lesions in the menisci, cartilage, bone marrow, or ligaments (Schueller-Weidekamm et al. 2006). Our results are in agreement with the latter, suggesting that sex differences in OA-related knee abnormalities in athletes are not yet evident at these relatively young ages.

Osteophytes were found in 39 and 56% of the adolescents at BL and FU respectively, and in 94 and 100% of the adults at those time points. The prevalence of osteophytes in the adults is higher than the 74% prevalence rate of osteophytes in an MRI study of 710 healthy adults with a mean age of 62.3 years (Guermazi et al. 2012). A previous study in 685 subjects with a mean age of 28.5 years found that osteophytes were more prevalent in athletes and those who had undergone surgery (Roemer et al. 2015), suggesting that both long-term volleyball participation and prior knee surgeries contributed to the high prevalence of osteophytes in our adult cohort, as well as their greater prevalence and severity of osteophytes compared to those found in adolescents. At FU, adult athletes with prior knee injuries had more severe osteophytes than those without prior knee injury, which corresponds well to previous literature showing injury status to be a predictor of knee OA (Kohatsu and Schurman 1990, Felson et al. 2000). Since osteophytes are a hallmark of and are associated with structural progression of OA (Audrey et al. 2014, Barr et al. 2015), it would be fruitful for further studies to investigate how they correlate with the symptoms and development of OA in athletes in the long-term.

Several of the volleyball athletes demonstrated BMLs, which consist of focal signals of abnormality in the subchondral bone marrow and are believed to be caused by capillary leakage caused by trauma, lesions, or increased intravascular pressure due to either increased blood flow to or decreased venous clearance of the marrow space (Eriksen and Ringe 2012). Previous studies have found BMLs in asymptomatic athletes: Soder et al. found that 50% of 28 asymptomatic 14- to 15-year old soccer players had BMLs (Soder et al. 2011), and Major et al. found that 41% of 17 varsity college basketball players had BMLs (Major and Helms 2002). In comparison, only 22 and 17% of the adolescent volleyball players in our study showed BMLs at BL and FU respectively. However, 44 and 61% of the adult volleyball athletes showed BMLs at BL and FU, which is comparable to previous studies and may be due to their higher age and longer-term participation in volleyball. It has been suggested that continuous repetitive jumping and running may explain the common finding of BMLs in athletes (Kornaat et al. 2008). In another study, persistent participation in vigorous physical activity was associated with worsening cartilage changes in the medial compartment in healthy adults with BMLs, but not in those without BMLs (Teichtahl et al. 2012). For future studies, it would be valuable to compare knee abnormalities between athletes and non-athletes to distinguish the effect of physical activity participation from age on BMLs.

Subarticular cysts were found in 11% of adolescent and 28% of adult volleyball players at both BL and FU. Similarly, the study by Soder et al. found cysts in 11% of asyptomatic adolescent soccer players (Soder et al. 2011), and Guermazi et al. found subarticular cysts in 19% of healthy adults aged between 50 and 60 years old (Guermazi et al. 2012). This suggests that volleyball athletes do not have a higher prevalence of subarticular cysts than the general population. Since subarticular cysts are usually asymptomatic (Audrey et al. 2014), those found in our cohort are unlikely to have any clinical significance. Additionally, Audrey et al. found that subarticular cysts were present in only 30.6% of 806 knees with radiographic OA, and concluded that they may be a late pathological feature of knee OA, but should not be considered a cardinal radiographic feature of OA (Audrey et al. 2014).

Meniscal lesions were found in 28% of the adoelscents at BL and FU, and in 56 and 61% of the adults at BL and FU, respectively. Soder et al. found no meniscal lesions in asymptomatic adolescent soccer players; however, this may be because they utilized low-field MRI (0.35-T), which is less sensitive than the high-field unit (1.5-T) utilized in this study to detect such lesions. Previous studies have had mixed findings of the prevalence of meniscal lesions in adult athletes, including 0% in varsity college basketball players (Major and Helms 2002), 20% in professional basketball players (Kaplan et al. 2005), and 58% in professional football players (Reinig et al. 1991). The high prevalence found in our adult cohort is largely attributable to the fact that 5 adults (28%) had prior meniscectomies; excluding those cases, 28 and 33% of adults had meniscal lesions at BL and FU, which is comparable to past findings. The greater severity of meniscal lesions in adults than in adolescents is also likely due to the high rate of meniscectomies in that age group. Meniscal lesions have been shown to lead to early-stage knee OA (Englund et al. 2009), so future studies should assess whether meniscal lesions in adolescent and adult athletes correlate with OA in the long-term.

There were several limitations to this study. There was a small sample size, so results must be considered preliminary until confirmed in larger cohorts. There was also no non-athletic control group, and there was no radiographic data for comparison. This study also had several strengths. The subjects were all highly-active volleyball players, allowing for comparison of age and sex cohorts with similar activity levels in the same high-impact sport. We utilized MRI, which provides advantages over radiography through its ability to assess articular tissues, including cartilage, menisci, and ligaments, and is thus most well-suited as a tool for whole-organ joint imaging (Peterfy et al. 2004).

## Conclusions

In conclusion, we found significant differences in the prevalence and severity of knee abnormalities between adolescent and adult volleyball players, but no differences by sex. These findings agree with previous MRI studies of knee abnormalities in adolescent and adult athletes (Reinig et al. 1991, Major and Helms 2002, Kaplan et al. 2005, Soder et al. 2011), but is the first study to cross-sectionally and longitudinally examine these characteristics in volleyball athletes. These results lay the groundwork for further investigations with larger cohorts and longer FU times to determine the long-term effects of knee pathology in athletes of high-impact sports, and whether or not they are associated with the development of OA. These results support the role of MRI in aiding clinical diagnosis and in the prevention and treatment of injuries in athletes.
